# Autism, Early Psychosis, and Social Anxiety Disorder: a transdiagnostic examination of executive function cognitive circuitry and contribution to disability

**DOI:** 10.1038/s41398-018-0193-8

**Published:** 2018-09-24

**Authors:** Eleni A. Demetriou, Christine Y. Song, Shin H. Park, Karen L. Pepper, Sharon L. Naismith, Daniel F. Hermens, Ian B. Hickie, Emma E. Thomas, Alice Norton, Django White, Adam J. Guastella

**Affiliations:** 1 Autism Clinic for Translational Research, Brain and Mind Centre, Central Clinical School, Faculty of Medicine, University of Sydney, Camperdown, 2050 Australia; 2 Youth Mental Health Unit, Brain and Mind Centre, Central Clinical School, Faculty of Medicine, University of Sydney, Camperdown, 2050 Australia; 3 School of Psychology, University of Sydney, Camperdown, 2050 Australia

## Abstract

The disability burden in clinical cohorts with social impairment is significant, leading to poor functional outcomes. Some of this impairment has been linked to executive dysfunction. In this study, a transdiagnostic approach was taken to identify executive function (EF) processes in young adults that may underpin social impairment and to evaluate their contribution to disability. Comparisons were made between three prominent disorders that are characterized by social impairments, Autism Spectrum Disorder (ASD), Early Psychosis (EP) and Social Anxiety Disorder (SAD), as well as a neurotypically developing group (TYP). We examined whether overall disability could be predicted by neuropsychological and self-report assessments of EF. Our study showed that ASD participants demonstrated impaired performance on most domains of EF compared to the TYP group (mental flexibility, sustained attention and fluency) while the EP group showed impairment on sustained attention and attentional shifting. The SAD participants showed EF impairment on self-report ratings, even though their objective performance was intact. Self-reports of EF explained a significant percentage (17%) of disability in addition to the variance explained by other predictors, and this was particularly important for ASD. This is the first study to compare EF measures across clinical groups of social impairment and suggests unique cognitive-circuitry that underpins disability within groups. Impairments in EF were broad in ASD and predicted disability, EP impairments were specific to attentional processes and SAD impairments likely relate to negative self-monitoring. Self-report, as opposed to performance-based EF, provided best capacity to predict disability. These findings contribute to transdiagnostic circuitry models and intervention strategies.

## Introduction

The disability burden in clinical cohorts with social impairment is significant and typically associated with poor functional outcomes^1–3^. Social impairment and disability have been linked to poor executive function (EF) performance^4,5^ in these groups and EF may be a useful cognitive marker to predict disability. Assessment of EF is traditionally based on neuropsychological (objective) measures of the level of performance across cognitive domains^6^. More recently, standardised scales of self/informant (subjective) based ratings of EF have been introduced with empirical support that these may be more ecologically valid assessments of EF^7^. Understanding how EF and underlying cognitive circuitry may contribute to disability in clinical groups with social impairment and, more specifically, ascertaining the contributions of objective and subjective measures of EF may be pivotal for diagnosis and functional outcomes. Such research is particularly important in early adulthood^8^ when brain development and transition into higher education, work, and adult social relationships coincide with establishing lifelong roles.

Autism Spectrum Disorder (ASD), Psychotic Disorders and Social Anxiety Disorder (SAD) are the three most common and recognised conditions characterized by social impairment. Traditionally, the influence of EF has been examined within disorders. Numerous researchers have proposed common aetiologies and maintaining factors underpinning disability in these cohorts, raising potential for common circuitry processes across psychiatric disorders that may predict disability^9,10^. In the neurodevelopmental cluster (i.e. ASD, Psychosis) EF is believed to result from differential brain development^11^ which may contribute to disability^12^. Empirical support for a link between EF and social impairment has been reported for ASD^4,13^ and psychotic disorders^14^, including the most common psychotic presentation in young adults, Early Psychosis (EP). These EF impairments may contribute to poor functional outcomes^14,15^. Neural circuitry studies show involvement of the prefrontal cortex (PFC) and, in particular, the dorsolateral and ventrolateral prefrontal cortex (DLPFC, VLPFC) in EF performance for both schizophrenia and ultra-high risk psychotic populations^16,17^. Within ASD, a recent meta-analysis suggested more global executive dysfunction^18^ with little evidence of selective dysfunction in specific EF domains (although few studies examined adult samples). Similarly, the underlying brain circuitry in ASD suggests widespread functional and anatomical differences^19^, although neuroimaging studies have also identified specific deficits in the PFC^20^ and fronto-striatal circuitry^21^. To date however, the relationship of disability to EF has been explored by few studies^22^ and mostly in children or young adolescents^13,23^. There has also been no transdiagnostic examination of the influence of EF to disability in neurodevelopmental cohorts.

Where the primary presenting condition is SAD, the relationship between neuropsychological difficulties and disability is more tenuous^5,24^. Mixed results have noted potential deficits in mental flexibility, verbal fluency^24^ and in the areas of sustained attention and concept formation^5^. There is no clear empirical support, however, for impaired neural circuitry specific to EF in SAD. For SAD, underlying aetiology and maintaining factors are believed to focus primarily on fear circuitry^25^, maladaptive cognitions, negative evaluation^26^ and avoidance^27^. Research suggests that these may be modulated by impaired top-down connectivity between the ventromedial prefrontal cortex (VMPFC), the amygdala^28^ and aberrant processing in the default mode network (DMN), leading to disturbed self-evaluative and self-referential processing^28^. Significant impairment in social functioning has been reported in SAD^27^ however, the relationship between EF and disability in SAD has not been specifically studied.

### Study aims

In this study, we aim to address the relationship between EF and disability in young adults with disorders characterized by social impairment. As far as we are aware, no study has yet compared EF performance across these disorders. The first aim of this study was to determine EF outcomes in treatment seeking young adults with presenting primary diagnosis of ASD, SAD and EP, and a neurotypical control group. It was predicted that young adults diagnosed with ASD and EP would show broad EF impairments in comparison to those with SAD and neurotypical controls. The second aim of this study was to evaluate the predictive value of EF to disability across and within these cohorts. It was predicted that EF would predict disability for participants with ASD and EP, but not SAD. Finally, we were interested in the degree that performance measures or self-report measures predicted disability.

## Method

### Participants

The University of Sydney Ethics Committee approved the research protocol (No. 2013/352) for this project and informed consent was obtained directly from each participant prior to inclusion in the study. A cohort of young adults (*N* = 253; Age: M = 23.16, SD = 5.80) presenting for treatment and/or social skills development at the Autism Clinic for Translational Research (ACTr) and *Headspace* Brain and Mind Centre clinics, were recruited into the study. Participants met criteria for the primary presenting disorder of either ASD, *N* = 60; EP, *N* = 58; or SAD, *N* = 76. Clinical diagnoses were made by qualified clinicians at the ACTr, based on standardised clinical interviews and diagnostic assessment instruments. Neurotypical control participants (TYP; *N* = 59) were recruited through advertising at a number of different university websites. All participants were screened and excluded from the study if they had intellectual disability (IQ < 70) or current substance dependence. The TYP participants were excluded if they reported a mental health diagnosis (past or current), were currently experiencing significant levels of depression, anxiety or stress (DASS-21), or if they scored at clinical cut-offs for SAD (SIAS) and/or ASD (AQ).

### Measures

A detailed description of all measures is provided in Supplementary Table 1. The assessment battery comprised of a combination of diagnostic assessments, neuropsychological tests and self-report measures of EF, mood and disability. Diagnostic assessments consisted of standardised clinical interviews for the diagnosis of ASD (ADOS), EP (SCID-I) and SAD (ADIS), psychotic symptoms were assessed with the SAPS and SANS scales. Neuropsychological tests comprised of assessments of pre-morbid IQ (WTAR) and performance measures of EF assessing the domains of ‘Set Shifting’ (CANTAB-IED), ‘Mental Flexibility’ (TMT-B) and ‘Verbal Fluency’ (COWAT). The cognitive domains of ‘Sustained Attention’ (CANTAB-RVP) and ‘Psychomotor Speed’ (TMT-A) were also assessed. Self-report ratings of EF and disability were based on the BRIEF and WHODAS respectively. Symptom severity was assessed by the DASS-21, AQ and the SIAS.

### Data analysis

Data analysis was performed using the IBM SPSS Statistics version 24. Univariate (ANOVA) and Multivariate Analysis of Variance (MANOVA) examined differences between the groups on EF and disability. Two-sided tests with Bonferroni confidence interval adjustments were made for all statistical comparisons, alpha was set at *p* = 0.01 to control for multiple comparisons. Multiple Regression (MR) analyses examined the predictive value of the EF measures on disability. The EP group did not complete the BRIEF and MR analysis for this group were based on performance only measures of EF. For each of the MR analyses, IQ, Education, Depression and EF performance measures were entered in Step 1 and where available, the BRIEF was entered in Step 2.

## Results

### Statistics

Sample size was based on the number of participants tested at the conclusion of the study. Our sample size per group exceeded the suggested minimum (*n* = 30)^29^ for violations against the assumptions of normality and equality of variance in MANOVA. The MANOVA was carried out with and without the two covariates (IQ, Education) and the statistical outcomes were comparable. Results are reported for the analysis including covariates. All assumptions for MR were met with the exception of multivariate outliers as assessed by Mahalanobis Distance. An examination of the largest outliers together with the Statistic for Cook’s Distance indicated that they do not exert an influence on the model^29^ and no further action was taken.

### Demographics

Participant demographic information is presented in Table 1. No significant gender differences were observed between the diagnostic and control groups, *χ*^2^_(3, *N*=253_) = 2.73, *p* > 0.05. The overall significant effect for Age (*F*_3, 249_ = 4.33, *p* = 0.005) did not hold for pairwise comparisons. Significant overall effects were observed for Education (*F*_3,249_ = 13.85, *p* < 0.001), IQ (*F*_3,249_ = 18.48, *p* < 0.001) and Depression (*F*_3,224_ = 34.09, *p* < 0.001). Pairwise comparisons showed that: the TYP group had significantly more years of education compared to each of the clinical groups; the EP group had significantly lower IQ compared to each of the other groups. The clinical groups reported higher levels of depression compared to the TYP group with no differences between the clinical groups.

**Table 1:** Demographic characteristics and participants responses to the Depression Anxiety Stress Scale (DASS)

	ASD_a_		EP_b_		SAD_c_		TYP_d_		Statistical analysis						
Gender	*N*	%	*N*	%	*N*	%	*N*	%	Pearson Chi-Square						
Male	38	63.3	37	63.8	41	53.9	31	52.5	*χ*^2^_(3, *N*=253)_ = 2.73, *p* > 0.05						
Demographic characteristics	Mean	SD	Mean	SD	Mean	SD	Mean	SD	ANOVA	Post-hoc Multiple Comparisons with Bonferroni adjustment (*p* = 0.01)					
*N*	60		58		76		59			a vs b	a vs c	a vs d	b vs c	b vs d	c vs d
Age (years)	24.11	7.27	21.79	4.05	22.11	5.64	24.88	5.3	*F*_(3,249)_ = 4.33*	ns	ns	ns	ns	ns	ns
Education (years)	12.43	1.96	12.24	2.2	12.61	1.84	14.37	2.19	*F*_(3,249)_ = 13.85**	ns	ns	**	ns	**	**
IQ	107.56	8.66	100.71	9.37	111.28	6.56	106.99	8.3	*F*_(3,249)_ = 18.48**	**	ns	ns	**	**	ns
Depression Anxiety Stress Scale - DASS-21	Mean	SD	Mean	SD	Mean	SD	Mean	SD	ANOVA	Post-hoc multiple comparisons with Bonferroni adjustment (*p* = 0.01)					
*N*	55		49		65		59			a vs b	a vs c	a vs d	b vs c	b vs d	c vs d
DASS 21 _Depression_	19.38	13.44	17.38	12.24	22.77	12.03	3.73	4.83	*F*_(3,224)_ = 34.09**	ns	ns	**	ns	**	**

*p ≤ 0.01

**p < 0.001

### Group comparison of EF performance/self-report measures and disability self-report measures

**Fig. 1:**
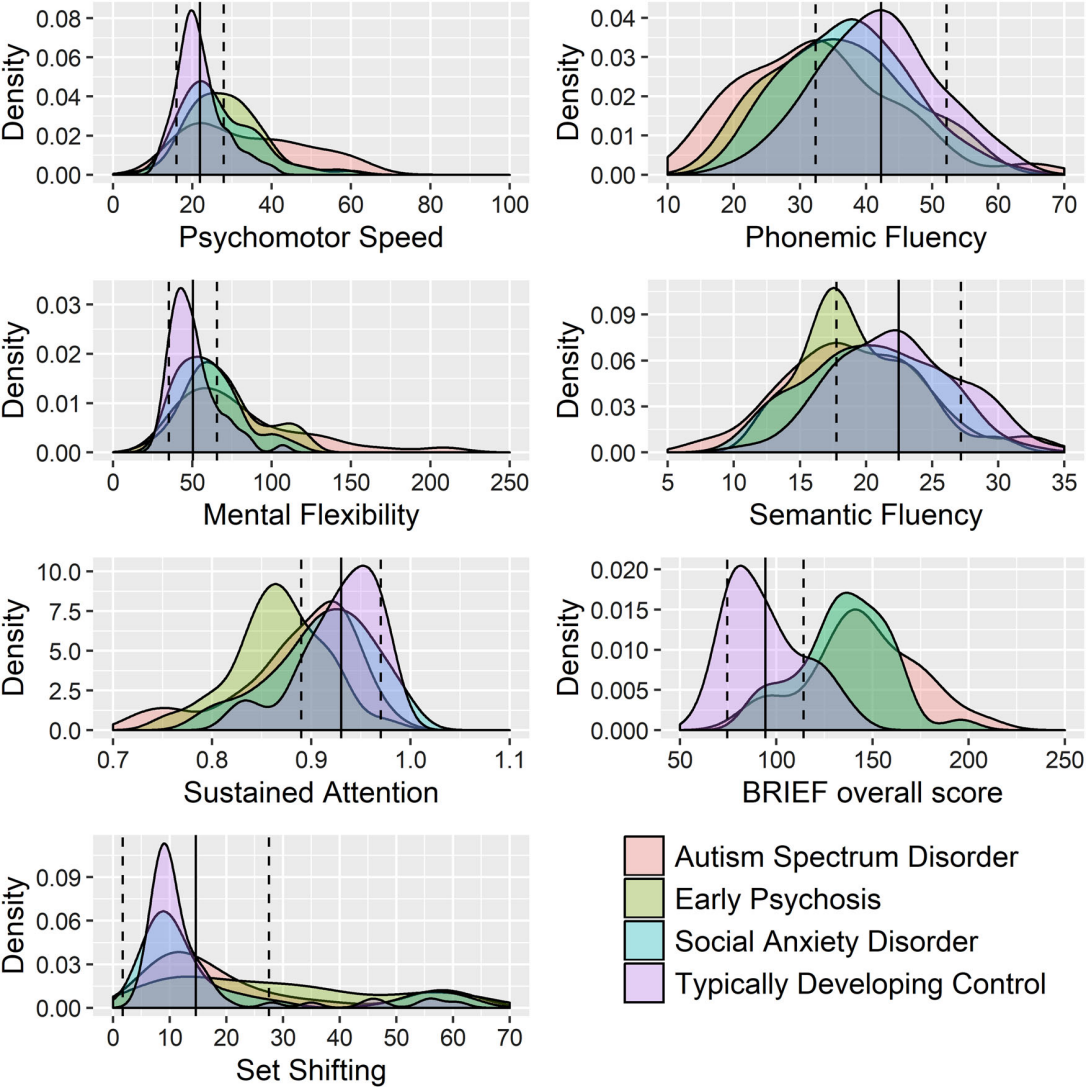
Response distribution of EF neuropsychological and self-report measures

For the distribution of participant responses for the neuropsychological and self-report measures of EF refer Fig. 1. Participants’ responses on all measures are summarised in Table 2.

**Table 2:** Group characteristics of executive function neuropsychological and self-report measures and disability self-report ratings

	ASD_a_		EP_b_		SAD_c_		TYP_d_		Statistical analysis						
**Neuropsychological measures**	**Mean**	**SD**	**Mean**	**SD**	**Mean**	**SD**	**Mean**	**SD**	**MANOVA**	Post-hoc multiple comparisons with Bonferroni adjustment (*p* = 0.01)					
	***N*** = **60**		***N*** **=** **58**		***N*** **=** **76**		***N*** **=** **59**			**a vs b**	**a vs c**	**a vs d**	**b vs c**	**b vs d**	**c vs d**
TMT-A	35.61	19.37	28.41	9.22	26.67	9.58	21.94	5.96	*F*_(3,247)_ = 11.37**	**	**	**	ns	ns	ns
TMT-B	80.38	40.74	72.95	38.64	60.47	19.9	50.45	15.15	*F*_(3,247)_ = 7.46**	ns	*	**	ns	ns	ns
RVP-A	0.88	0.06	0.87	0.05	0.91	0.05	0.93	0.04	*F*_(3,247)_ = 9.37**	*	ns	**	ns	**	ns
IED_Total errors_	27.04	27.88	35.61	32.14	19.08	18.07	14.62	12.89	*F*_(3,247)_ = 4.34*	ns	ns	ns	ns	*	ns
COWAT_Phonemic_	32.72	11.58	36.09	10.14	37.28	9.27	42.29	9.91	*F*_(3,247)_ = 7.09**	ns	ns	**	ns	ns	ns
COWAT_Semantic_	19.33	5.42	19.29	4.17	20.49	4.86	22.47	4.71	*F*_(3,247)_ = 3.90**	ns	ns	*	ns	ns	ns

Self-report measures	Mean	SD	Mean	SD	Mean	SD	Mean	SD	ANOVA^i^/MANOVA^ii^	Post-hoc multiple comparisons with Bonferroni adjustment (*p* = 0.01)					
BRIEF	*N* = 48		n/a		*N* = 35		*N* = 30			a vs b	a vs c	a vs d	b vs c	b vs d	c vs d
BRIEF_GEC_^i^	144.79	28.98			135.54	23.62	94.33	19.8	*F*_(2,108)_ = 20.05**	n/a	ns	**	n/a	n/a	**
BRIEF_Inhibit_^ii^	15.35	3.81			14.4	3.29	10.97	2.33	*F*_(2,108)_ = 7.32**	n/a	ns	**	n/a	n/a	ns
BRIEF_Shift_^ii^	13.6	3.03			11.6	3.06	11.53	3.57	*F*_(2,108)_ = 19.50**	n/a	ns	**	n/a	n/a	**
BRIEF_Emotional Control_^ii^	20.96	5.78			19.49	5.61	13.4	3.63	*F*_(2,108)_ = 11.48**	n/a	ns	**	n/a	n/a	**
BRIEF_Self Monitor_^ii^	12.25	3.14			9.4	2.4	7.63	1.88	*F*_(2,108)_ = 17.72**	n/a	**	**	n/a	n/a	ns
BRIEF_Initiate_^ii^	17.6	4.28			17.69	3.09	11	2.59	*F*_(2,108)_ = 22.37**	n/a	ns	**	n/a	n/a	**
BRIEF_Working Memory_^ii^	16.79	4.36			16.06	4.04	10.53	2.91	*F*_(2,108)_ = 12.95**	n/a	ns	**	n/a	n/a	**
BRIEF_Plan Organize_^ii^	20.79	4.82			20.43	4.46	13.27	3.31	*F*_(2,108)_ = 14.71**	n/a	ns	**	n/a	n/a	**
BRIEF_Task Monitor_^ii^	11.9	2.65			12.11	2.41	8.4	1.96	*F*_(2,108)_ = 11.72**	n/a	ns	**	n/a	n/a	**
BRIEF_Organization Materials_^ii^	15.54	4.27			14.94	3.96	11.3	2.69	*F*_(2,108)_ = 5.79**	n/a	ns	*	n/a	n/a	ns
**WHODAS**	***N*** **=** **54**		***N*** **=** **50**		***N*** **=** **62**		***N*** **=** **58**			**a vs b**	**a vs c**	**a vs d**	**b vs c**	**b vs d**	**c vs d**
WHODAS_Total_^i^	35.59	18.67	37.26	15.84	38.01	16.18	7.51	6.91	*F*_(3,218)_ = 42.23**	ns	ns	**	ns	**	**
WHODAS_Domain 1_^ii^	39.74	22.74	40.18	18.17	40.21	18.89	10.52	11.87	*F*_(3,218)_ = 26.77**	ns	ns	**	ns	**	**
WHODAS_Domain 2_^ii^	21.88	21.29	24.88	21.89	18.25	21.18	2.37	5.59	*F*_(3,218)_ = 10.73**	ns	ns	**	ns	**	**
WHODAS_Domain 3_^ii^	18.64	21.78	15.87	16.89	13.87	16.63	1.38	3.95	*F*_(3,218)_ = 8.39**	ns	ns	**	ns	**	**
WHODAS_Domain 4_^ii^	49.81	24.08	40.54	27.85	58.57	22.84	10.63	14.29	*F*_(3,218)_ = 43.34**	ns	ns	**	**	**	**
WHODAS_Domain 5_^ii^	40.19	31.71	40.2	28.1	42.1	26.44	10	14.51	*F*_(3,218)_ = 14.56**	ns	ns	**	ns	**	**
WHODAS_Domain 6_^ii^	35.87	25.51	44.85	18..18	42.35	21.46	6.25	9.57	*F*_(3,218)_ = 37.55**	ns	ns	**	ns	**	**

*TMT-A* Trail Making Test A, *TMT-B* Trail Making Test B, *RVP* Rapid Visual Processing, *IED* Intra/Extra Dimensional Shift, *COWAT* Controlled Oral Word Association Test, *BRIEF* Behavioral Rating Inventory of Executive Function, *WHODAS* WHO Disability Assessment Scale

*p ≤ 0.01

**p ≤ 0.001

For the corrected model, a significant overall MANOVA effect was observed for the overall diagnosis (Hotelling’s *T*: *F* = 4.23, *p* < 0.001). Follow-up analyses showed significant overall effects for the domains of Psychomotor Speed (*F*_3,247_ = 11.37, *p* < 0.001), Mental Flexibility (*F*_3,247_ = 7.46, *p* < 0.001), Sustained Attention (*F*_3,247_ = 9.37, *p* < 0.001), Set Shifting (*F*_3,247_ = 4.34, *p* = 0.005), Phonemic Fluency (*F*_3,247_ = 7.09, *p* < 0.001) and Semantic Fluency (*F*_3,247_ = 3.90, *p* = 0.009).

Post-hoc pairwise comparisons indicated that the ASD group showed the greatest level of EF impairments. The ASD group performed significantly worse compared to at least one or more of the other groups on the domains of: Psychomotor Speed (ASD < EP, ASD < SAD and ASD < TYP); Mental Flexibility (ASD < TYP and ASD < SAD); Sustained Attention (ASD < TYP); Phonemic Fluency (ASD < TYP) and Semantic Fluency (ASD < TYP). The EP group was significantly impaired on Sustained Attention (EP < ASD, EP < TYP) and on Set Shifting (EP < TYP). The SAD group showed intact EF on all performance measures.

The ANOVA analysis comparing differences between groups on the BRIEF (overall score) was significant (*F*_2,108_ = 20.05, *p* < 0.001). Pairwise comparisons revealed that both ASD and SAD groups reported similar and significantly higher levels of EF impairment compared to the TYP group. The overall MANOVA analysis for Diagnosis was significant for the BRIEF clinical scales (Hotelling’s *T*: *F* = 5.63, *p* < 0.001). Pairwise comparisons showed that the ASD group reported significantly impaired EF on all clinical scales compared to the TYP group and on the Self-Monitor subscale compared to the SAD group. The SAD group also reported significant impairment on most clinical scales compared to the TYP group. The non-significant results were for the scales of Inhibit, Self-Monitor and Organization of Materials.

A significant overall ANOVA effect was observed for Diagnosis for the WHODAS (overall score) (*F*_3,218_ = 42.23, *p* < 0.001). Pairwise comparisons indicated that each of the clinical groups reported significantly higher levels of overall disability compared to the TYP group with no differences between the clinical groups. The overall MANOVA analysis of the WHODAS domains was also significant (Hotelling’s *T*: *F* = 10.71, *p* < 0.001). Each of the clinical groups reported significant impairment compared to the TYP group for each of the six WHODAS domains, in addition the SAD group demonstrated significantly higher levels of impairment on the ‘Getting along’ domain (Domain 4) compared to the EP group.

### Effects of EF neuropsychological predictors on disability

The first MR examined the relationship of the predictors of Diagnosis, EF performance measures, IQ, Education and Depression on disability (Table 3). The model for EF performance measures and disability was significant across the study cohort (*F*_10,209_ = 37.11, *p* < 0.001) and explained 64.0% of the total variance, with significant predictors Education (*β* = −0.169, *p* < 0.001) and Depression (*β* = 0.726, *p* < 0.001). As Diagnosis was not a significant predictor no follow up interaction models were examined.

**Table 3:** Effects of EF neuropsychological predictors on disability

	*B*	SE *B*	*β*
Constant	49.673	20.081	
Diagnosis	0.662	0.882	0.036
Education	−1.521	0.414	−0.169**
IQ	0.044	0.112	0.020
TMT-A	0.176	0.088	0.114
TMT-B	−0.035	0.035	−0.057
RVP-A	−25.174	19.108	−0.071
IED_Total_ _errors_	0.010	0.037	0.012
Fluency_Phonemic_	−0.068	0.100	−0.037
Fluency_Semantic_	−0.093	0.209	−0.023
DASS_Depression_	1.078	0.068	0.726**

**p < 0.001

### Additive effect of EF self-report predictor on disability

A second regression analysis was completed for the participants that completed the self-report measure of EF (excludes EP group). The procedure outlined above was followed for Step 1 and the self-report measure (BRIEF) was entered in Step 2 (Table 4). Model 1 accounted for 64.0% of the total variance (*F*_10,97_ = 17.22, *p* < 0.001) with the only significant predictor Depression (*β* = 0.726, *p* < 0.001). When the BRIEF was entered in Step 2 (method Enter), Model 2 accounted for an additional 17.0% of the variance (*F*_1,96_ = 86.14, *p* < 0.001) with the following significant predictors: Diagnosis (*β* = 0.583, *p* < 0.001), Education (*β* = 0.161, *p* = 0.009), IQ (*β* = 0.227, *p* < 0.001), IED_Total errors_ (*β* = −0.146, *p* = 0.007), Depression (*β* = 0.325, *p* < 0.001) and BRIEF (*β* = 0.967, *p* < 0.001). A follow-up analysis was performed to examine the interaction of diagnosis with the five significant predictors (Diagnosis × Education, Diagnosis × IQ, Diagnosis × IED_Total errors_, Diagnosis × Depression and Diagnosis × BRIEF). Depression was a significant predictor of disability for the ASD (*B* = 0.621, *p* < 0.001) and the SAD (*B* = 0.711, *p* < 0.001) groups. The BRIEF significantly predicted disability for the ASD group only (*B* = 0.248, *p* = 0.001). None of the other interaction comparisons were significant.

**Table 4:** Additive effect of EF self-report predictor on disability

	*B*	SE *B*	*β*
**Step 1**			
Constant	49.673	29.476	
Diagnosis	0.662	1.295	0.036
Education	−1.521	0.607	−0.169
IQ	0.044	0.164	0.020
TMT-A	0.176	0.129	0.114
TMT-B	−0.035	0.052	−0.057
RVP-A	−25.174	28.048	−0.071
IED_Total_ _errors_	0.010	0.055	0.012
Fluency_Phonemic_	−0.068	0.147	−0.037
Fluency_Semantic_	−0.093	0.306	−0.023
DASS_Depression_	1.078	0.099	0.726**
**Step 2**			
Constant	−119.902	28.223	
Diagnosis	10.578	1.426	0.583**
Education	1.456	0.547	0.161*
IQ	0.500	0.130	0.227**
TMT-A	−0.152	0.101	−0.099
TMT-B	0.073	0.039	0.120
RVP-A	−16.501	20.490	−0.046
IED_Total_ _errors_	−0.117	0.042	−0.146*
Fluency_Phonemic_	−0.286	0.110	−0.155
Fluency_Semantic_	−0.188	0.224	−0.047
DASS_Depression_	0.482	0.097	0.325**
BRIEF	0.585	0.063	0.967**

*p < 0.01, **p < 0.001

## Discussion

This study is the first to compare underlying cognitive markers for three disorders characterised by social impairment, EP, SAD, and ASD, and specifically evaluate EF and its role in predicting disability. Our study showed that, despite no apparent impairments in intellectual function, ASD participants showed significant and broad impairments across the domains of Mental Flexibility, Sustained Attention and Fluency, as well as Psychomotor Speed. Participants with EP showed impairments on Sustained Attention and Set Shifting. Interestingly, despite adequate EF performance on the objective measures, SAD participants self-reported EF impairment that was of a similar degree to those diagnosed with ASD. Our findings suggest that different cognitive-circuitry is associated with social impairment in these cohorts and in particular, broad EF connectivity^19^ in ASD, attentional switching^17^ in EP and self-referential processes^28^ in SAD. Further research is now required linking the neurobiological underpinnings of these to this disability.

The second aim of this study was to establish which EF measures predicted disability. Results showed that the self-report measure of EF and depression predicted disability overall. The relationship between depression and disability was significant across both the ASD and SAD groups however, the self-report measure of EF explained additional proportion of the disability variance for the ASD group only. This study therefore provides evidence that deficits in EF are particularly pronounced in ASD and may be particularly important in terms of predicting disability.

For the ASD participants, the majority of EF performance measures differentiated these participants from the TYP group. While we acknowledge there was variability even within the ASD group itself, this study supports assertions of broad executive dysfunction in young adults with ASD that are likely neuro-developmentally driven. We have previously argued that broad connectivity models of brain development, rather than region specific brain processes, underpin these impairments^18^. Interestingly, the EP group performed significantly worse than the TYP group on the RVP and IED, tests which purport to assess attentional processes^30^. This finding is in line with involvement of the DLPFC^31^. These domains have previously been shown to predict conversion of clinical high risk individuals to psychosis^32^ and may reflect a higher level of impairment. The lack of any further EF impairment in this group supports empirical findings that broad EF in early stages of psychosis remains intact^33^. There was no evidence of EF impairment in SAD on neuropsychological measures, which was expected given that the underlying aetiology for SAD is focused on fear circuits^25^ and maladaptive cognitions^27^.

While we did not have data available for EP participants, both SAD and ASD participants reported similar and significant impairment on the EF self-report measure. This was despite the SAD group showing objective performance on all EF measures that was similar to neurotypical controls. A number of factors may contribute to this finding. Firstly, it may extend the adverse influence of maladaptive cognitions^34^ and self-focused attention^35^ beyond social performance to other areas of self-evaluation, including EF. That is, individuals with SAD negatively evaluate their own adaptive functioning in relation to EF. This might suggest that self-report measures of EF are significantly influenced by anxiety driven biases and exploration of the role of anxiety across all of these cohorts on EF self-report measures is required. Secondly, it may reflect the assertion that self-report ratings of EF assess a different cognitive construct that is not constrained by performance outcomes of objective cognitive measures, but instead is goal oriented and reflects the individual’s beliefs relevant to these goals^36^. This may be particularly pertinent in individuals with SAD given their intact performance on objective measures of EF and may augment reporting of executive dysfunction in ASD individuals. Partial support for this proposal was found by the observed low correlation between BRIEF domains and neuropsychological measures of EF^37^ (Supplementary Table S2), suggesting they might tap different cognitive processes. Alternatively, rating measures of EF may be more ecologically valid and thus better capture functional outcomes associated with EF^7^.

All of our young adult clinical groups presented to our specialist assessment clinics and reported significant disability compared to the neurotypical control group as measured by the WHODAS. Results showed that in addition to ‘Depression’, the self-report measure of EF was the strongest predictor of disability in this cohort but, within groups, this relationship was significant only for the ASD group. In contrast, neuropsychological measures of EF were largely unrelated to disability in all groups. The lack of relationship between EF and disability in the other clinical groups indicates that other factors may be more important including affective states^24^ and co-morbid conditions^38^. Further research is needed to elucidate the specific contribution of these factors to disability, which ultimately is linked to participation in society, educational and vocational outcomes^3,39^.

Our study has several limitations. Our performance measures of EF although broad did not encompass all commonly accepted subdomains of EF. We do note, however, our previous work that has highlighted the relative equivalence of EF domains in ASD and across development^18^. Further, the BRIEF was only completed for part of our cohort (ASD/SAD/TYP). Although our results on the relationship between the BRIEF and disability are quite robust, they rely on a self-report measure of disability. Further examination of these against objective measures of disability and on larger samples would further inform the significance of these findings.

## Conclusions

This is the first study to examine both objective and subjective markers of EF across three clinical groups of young adults with social impairment and to evaluate their relationship to disability. The study lends support to the importance of executive dysfunction in the ASD population both in differentiating between clinical groups and in self-reported EF predicting disability. It also indicates that EF deficits are not likely primary contributors to disability for SAD and EP, despite the SAD group reporting EF impairment on self-report measures and the EP group performing worse on tests of sustained attention and attentional shifting. The EF outcomes across our cohort support impaired self-referential processing that may relate to DMN processes in SAD, broad executive dysfunction that may be primarily driven by aberrant connectivity in ASD and, impaired attentional processes driven by the DLPFC in EP. Overall, these findings have treatment implications for young adults with social impairment and suggest that ASD therapeutic support may need to include cognitive training of EF, whereas in SAD and EP focus on maladaptive cognitions and attentional processes respectively, may be more appropriate.

## Electronic supplementary material

Supplementary tables S1 and S2
