# Supplementary material for: Autism, Early Psychosis, and Social Anxiety Disorder: a transdiagnostic examination of executive function cognitive circuitry and contribution to disability
Source: Transl Psychiatry. 2018 Sep 24;8:200. doi: 10.1038/s41398-018-0193-8 (PMC6155256; doi:10.1038/s41398-018-0193-8)
Supplement: Supplementary file 1 — Supplementary tables S1 and S2 [file 41398_2018_193_MOESM1_ESM.docx]

**Table S1. Clinical, neuropsychological and disability measures.**

| **Measure** | **Domain** | **Characteristics** |
| --- | --- | --- |
| **Clinical Measures** | | |
| ADOS-2  Autism Diagnostic Observation Schedule – 2nd edition^1^ | Autism Spectrum Disorder | Semi-structured, standardized assessment of:   - social interaction and communication, - restricted and repetitive behaviours |
| ADIS  Anxiety Diagnostic Interview Schedule^2^ | Social Anxiety Disorder | Semi-structured interview based on DSM-5 criteria for current assessment of mental health conditions and for differential diagnosis among them:   - anxiety - mood - obsessive-compulsive disorder - trauma - related disorders (e.g., somatic symptom, substance use) |
| SCID-I  The Structured Clinical Interview for DSM-IV Axis I Disorders^3^  SAPS  Scale for the Assessment of Positive Symptoms^4^  SANS  Scale for the Assessment of Positive Symptoms^4^ | First Episode Psychosis | Semi-structured interview for the major DSM-IV Axis I, II diagnoses. |
| **Neuropsychological measures** | | |
| WTAR  Wechsler Test of Adult Reading^5^ | Premorbid IQ |  |
| TMT-A  Trail Making Test-A^6^ | Psychomotor speed | Outcome measure is completion time in seconds, a higher score indicates worse performance |
| TMT-B  Trail Making Test-B^6^ | Mental flexibility | Outcome measure is completion time in seconds, a higher score indicates worse performance |
| COWAT  Controlled Oral Word Association Test^7^ | Phonemic and Semantic fluency | - Phonemic fluency, the letters “F”, “A”, and “S” were used - Semantic fluency, the “animal” category was used   Participants were required to name as many words as possible in each letter and animal categories within 60 seconds.  Outcome measures are total sum of words for the Phonemic and Semantic categories. A higher score indicates better performance. |
| IED  Intra-Extra Dimensional Shift Test^8^ | Set shifting and flexibility of attention | Outcome measures are:   - stages completed, the total number of stages completed successfully (range 1-9) - total errors (adjusted), the expected maximum number of errors irrespective of whether the participant completed all 9 stages (range 0-225). |
| RVP  Rapid Visual Processing Test^8^ | Sustained attention | Outcome measure is ‘RVP-A’, derived from Signal Detection Theory (SDT) and is a measure of sensitivity to the target (range 0.00-1.00). A score of ‘1’ indicates that the participant always detected the target. |
| **Self-report-measures** |  |  |
| BRIEF  Behavioural Rating Inventory of Executive Function^9^ | Executive function | A 75 item self-report questionnaire that assesses executive function. It consists of nine clinical scales that in combination provide an overall score of EF and two index scores:   - GEC - Global Executive Composite - BRI - Behavioural Regulation Index (derived from the clinical scales Inhibit, Shift, Emotional Control and Self Monitor) - MCI - Metacognition Index (derived from the clinical scales Initiate, Working Memory, Plan/Organize, Task Monitor, Organization of Materials) |
| WHODAS-2  World Health Organisation Disability Assessment Schedule-2^10^ | Disability | A 36-item self-report questionnaire that assesses the disability burden of mental and physical health problems overall and across six domains:   - Cognition - Mobility - Self-Care - Getting Along - Life Activities - Participation   The overall score range from 0-79. Higher scores indicate greater functional disability. |
| DASS-21  Depression Anxiety Stress Scale^11^ |  | A 21 item self-report questionnaire that assesses Depression, Anxiety and Stress over the last week.  Higher scores indicate greater impairment. |

**Table S2. Bivariate correlation between EF performance and self-report measures**

| Measure | 1 | 2 | 3 | 4 | 5 | 6 | 7 | 8 | 9 | 10 | 11 | 12 | 13 | 14 | 15 |
| --- | --- | --- | --- | --- | --- | --- | --- | --- | --- | --- | --- | --- | --- | --- | --- |
| 1. TMT-A | - |  |  |  |  |  |  |  |  |  |  |  |  |  |  |
| 2. TMT-B | .600** | - |  |  |  |  |  |  |  |  |  |  |  |  |  |
| 3. RVP-A' | -.465** | -.467** | - |  |  |  |  |  |  |  |  |  |  |  |  |
| 4. IED_Errors_ | .290** | .337** | -.350** | - |  |  |  |  |  |  |  |  |  |  |  |
| 5. Fluency_Phonetic_ | -.353** | -.315** | .339** | -.250** | - |  |  |  |  |  |  |  |  |  |  |
| 6. Fluency_Semantic_ | -.316** | -.330** | .287** | -.224** | .572** | - |  |  |  |  |  |  |  |  |  |
| 7. BRIEF_Inhibit_ | .350** | .213* | -.200* | .204* | -.120 | -.121 | - |  |  |  |  |  |  |  |  |
| 8. BRIEF_Shift_ | .336** | .361** | -.288** | .230* | -.245** | -.215* | .720** | - |  |  |  |  |  |  |  |
| 9. BRIEF_Emotional Control_ | .336** | .347** | -.299** | .395** | -.267** | -.134* | .609** | .776** | - |  |  |  |  |  |  |
| 10. BRIEF_Self Monitor_ | .441** | .366** | -.239* | .385** | -.238* | -.168 | .741** | .720** | .721** | - |  |  |  |  |  |
| 11. BRIEF_Initiate_ | .276** | .189* | -.167 | .081 | -.195* | -.164 | .736** | .702** | .540** | .518** | - |  |  |  |  |
| 12. BRIEF_Working Memory_ | .263** | .291** | -.229 | .155 | -.120 | -.151 | .810** | .798** | .603** | .584** | .796** | - |  |  |  |
| 13. BRIEF_Plan Organize_ | .328** | .258** | -.164 | .199* | -.153 | -.122 | .792** | .733** | .618** | .650** | .809** | .809** | - |  |  |
| 14. BRIEF_Task Monitor_ | .297** | .171 | -.185* | .105 | -.097 | -.096 | .739** | .643** | .506** | .515** | .775** | .807** | .806** | - |  |
| 15. BRIEF_Organization Materials_ | .210* | .123 | -.036 | .091 | -.119 | -.076 | .667** | .557** | .546** | .613** | .623** | .661** | .775** | .659** | - |

1. Lord C, Rutter M, DiLavore P, Risi S, Gotham K. *Autism diagnostic observation schedule-2nd edition (ADOS-2).* Los Angeles, CA.: Western; 2012.

2. Brown TA, DiNardo PA, Barlow DH. *Anxiety Disorders Interview Schedule for DSM-IV.* 198 Madison Avenue, New York, New York, 10016: Oxford University Press; 1994.

3. First MB, Gibbon M. The Structured Clinical Interview for DSM-IV Axis I Disorders (SCID-I) and the Structured Clinical Interview for DSM-IV Axis II Disorders (SCID-II). *Comprehensive handbook of psychological assessment, Vol. 2: Personality assessment.* Hoboken, NJ, US: John Wiley & Sons Inc; 2004:134-143.

4. Andresen NC, Flaum M, Arndt S, Alliger R, Swayze VW. *Positive and negative symptoms: Assessment and validity. Negative versus positive schizophrenia.* 1992.

5. Wechsler D. *Wechsler Test of Adult Reading.* San Antonio, Texas: Psychological Corporation; 2001.

6. Reitan RM, Wolfson D. *Trail Making Test: Manual for Administration and Scoring.* Tucson, AZ: Neuropsychological Press; 1985.

7. Lezak M, Howieson D, Bigler E, Tranel D. *Neuropsychological Assessment.* New York: oxford University Press; 2012.

8. De Luca CR, Wood SJ, Anderson V, et al. Normative Data From the Cantab. I: Development of Executive Function Over the Lifespan. *Journal of Clinical and Experimental Neuropsychology.* 2003;25(2):242-254.

9. Roth RM, Isquith PK, Gioia G. *BRIEF-A: Behavior Rating Inventory of Executive Function-Adult Version.* Lutz, FL 33549: PAR; 2005.

10. Ustun T, Kostanjsek N, Chatterji S, FRehm J. *Measuring health and Disability: Manual for WHO Disability Assessment Schedule, WHODAS 2.0.* Malta: World Health Organisation; 2010.

11. Lovibond PF, Lovibond SH. The structure of negative emotional states: Comparison of the Depression Anxiety Stress Scales (DASS) with the Beck Depression and Anxiety Inventories. *Behaviour Research and Therapy.* 1995;33(3):335-343.
